# Low-dose sulbactam-durlobactam for recurrent CRAB pneumonia in a child with cerebral palsy: a Case Report from a resource-limited setting

**DOI:** 10.3389/fphar.2026.1905442

**Published:** 2026-08-24

**Authors:** Mengting Li, Haotian Fei, Liang Huang, Lu Qing, Xiaoliang Liu, Guoyan Lu

**Affiliations:** 1 Department of Pediatrics, Ministry of Education Key Laboratory of Women and Children’s Diseases and Birth Defects, West China Second University Hospital, Sichuan University, Chengdu, Sichuan, China; 2 Department of Pediatrics, WCSUH-Tianfu·Sichuan Provincial Children’s Hospital, Meishan, Sichuan, China

**Keywords:** carbapenem-resistant *Acinetobacter* baumannii, low-dose regimen, OXA-23, pediatric pneumonia, resource-limited setting, sulbactam-durlobactam

## Abstract

**Background:**

Pediatric data for sulbactam-durlobactam (SUL-DUR) are extremely scarce, and no standard pediatric dosing exists.

**Methods:**

We report a 6-year-old boy with cerebral palsy and epilepsy who developed recurrent ventilator-associated pneumonia caused by OXA-23-producing carbapenem-resistant *Acinetobacter* baumannii (CRAB).

**Results:**

Due to financial constraints, a reduced-dose SUL-DUR regimen (150 mg/kg/day, q6h, 3-h infusion) was combined with cefoperazone-sulbactam to achieve a total sulbactam dose of 150 mg/kg/day. The patient defervesced within 48 h, inflammatory markers declined, chest imaging improved markedly, and he was transferred out of the PICU after 12 days of therapy.

**Conclusion:**

A PK/PD-driven low-dose SUL-DUR strategy may represent a feasible and potential cost-saving strategy for pediatric CRAB pneumonia in resource-limited settings.

## Introduction

*Acinetobacter* baumannii is a major cause of severe nosocomial infections, including ventilator-associated pneumonia, bloodstream infections, urinary tract infections, meningitis and wound infections, particularly in critically ill patients (Sharma and Lakhanpal, 2025; Moubareck and Halat, 2020). Nearly all major antibiotic classes have documented resistance in some A. baumannii strains, enabling the organism to survive for extended periods and disseminate widely in hospital settings (Müller et al., 2023). Resistance to carbapenems is primarily mediated by acquired OXA-type carbapenem-hydrolysing class D β-lactamases, with OXA-23 being the most common worldwide. The global CRAB population comprises at least nine clonal lineages, and isolates carrying blaOXA-23 predominate in most regions (Müller et al., 2023).

Current strategies to combat CRAB focus on optimized antibiotic combinations and emerging agents such as sulbactam-durlobactam (SUL-DUR; brand name *XACDURO*®) and cefiderocol (Makwana et al., 2026). SUL-DUR is a combination of sulbactam (a penicillin-derived β-lactam with intrinsic anti-Acinetobacter activity) and durlobactam (a diazabicyclooctane β-lactamase inhibitor) (McLeod et al., 2024). It was approved by the US FDA in May 2023 for the treatment of hospital-acquired and ventilator-associated bacterial pneumonia caused by susceptible *Acinetobacter* baumannii-calcoaceticus complex in adults (U.S. Food and Drug Administration, 2023).

To date, no pediatric pharmacokinetic data or completed clinical trials of SUL-DUR in children are available, and only a few case reports have been published. This report describes, to our knowledge, the first reported successful use of an individualized low-dose SUL-DUR strategy in a pediatric patient with chronic neurological comorbidities, providing preliminary real-world evidence for this vulnerable and understudied population.

### Case description

A 6-year-old boy with cerebral palsy and epilepsy (motor and language developmental delay) had been receiving long-term rehabilitation and antiseizure medication. Two months before the current admission, he was diagnosed with severe pneumonia and respiratory failure at a local hospital. Chest imaging showed bilateral infiltration and consolidation; electronic bronchoscopy revealed bilateral inflammation, tracheomalacia, laryngomalacia and abundant mucus plugs. Repeated sputum cultures, bronchoalveolar lavage fluid (BALF) cultures and metagenomic next-generation sequencing (mNGS) of BALF identified CRAB. He received sequential therapy with tigecycline, polymyxin B, cefoperazone-sulbactam, sulfamethoxazole-trimethoprim, minocycline and meropenem, after which he was successfully weaned from mechanical ventilation and transferred to rehabilitation.

**FIGURE 1:**
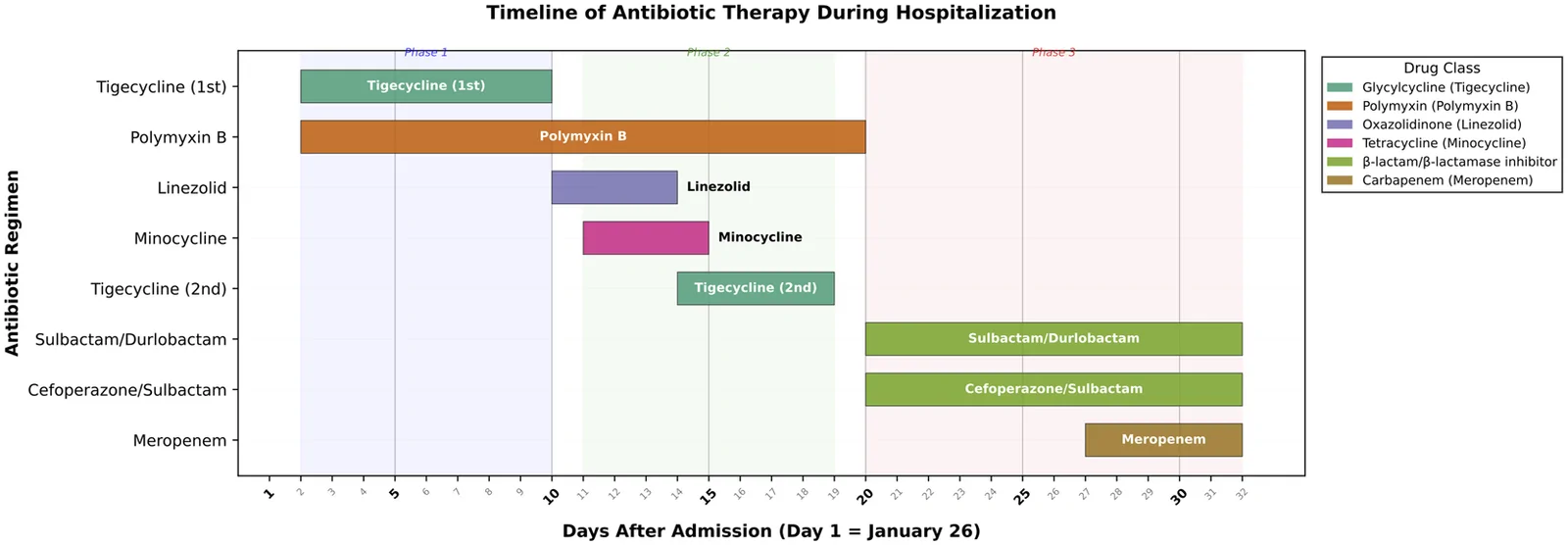
Timeline of antibiotic therapy during hospitalization.

During rehabilitation therapy, the patient developed recurrent respiratory distress with tachycardia, decreased oxygen saturation, and recurrent fever and was transferred back to the Pediatric Intensive Care Unit (PICU). Sputum culture again demonstrated CRAB (reported as abundant growth on semi-quantitative culture). Chest imaging revealed worsening bilateral infiltrates compared with prior studies. Antimicrobial susceptibility testing showed susceptibility to cefoperazone-sulbactam, sulfamethoxazole-trimethoprim, colistin, minocycline, and tigecycline; resistance to piperacillin-tazobactam, ceftazidime, cefepime, aztreonam, imipenem, meropenem, tobramycin, ciprofloxacin, doxycycline, piperacillin, and gentamicin; and intermediate susceptibility to levofloxacin. Of note, sulbactam-alone minimum inhibitory concentration (MIC) was not determined, as our clinical microbiology laboratory only performs susceptibility testing for the cefoperazone-sulbactam combination; the isolate was reported as susceptible to cefoperazone-sulbactam per CLSI criteria. Metagenomic next-generation sequencing of BALF identified *Acinetobacter* baumannii (2106 sequence reads) and detected the carbapenemase gene blaOXA-23, conferring resistance to carbapenems, penicillins, and cephalosporins. Collectively, fever (38.8 °C), tachycardia, hypoxaemia, worsening bilateral infiltrates, elevated CRP (50.37 mg/L), and high microbial burden (abundant growth on multiple cultures and 2106 mNGS reads) support true infection rather than colonization. Despite sequential combination therapy with tigecycline plus polymyxin B, minocycline and levofloxacin, the patient remained febrile, with persistently elevated inflammatory markers and worsening pulmonary infiltrates (Figure 1).

After consultation with the pharmacy department, sulbactam-durlobactam (SUL-DUR) was initiated on day 20 of admission. Due to severe economic constraints (the family could not afford the standard full-dose regimen), a reduced-dose regimen was adopted: SUL-DUR (sulbactam: durlobactam 1:1 fixed-ratio formulation) was initiated at 150 mg/kg/day, providing 75 mg/kg/day of sulbactam and 75 mg/kg/day of durlobactam, administered as a 3-h prolonged infusion every 6 h. The remaining 75 mg/kg/day of sulbactam was supplemented via cefoperazone-sulbactam, achieving a total sulbactam dose of 150 mg/kg/day. This dosing strategy was informed by the PK/PD profile of sulbactam, in which the time above MIC (%fT > MIC) is the primary driver of efficacy, and a prolonged 3-h infusion was selected to maximize this pharmacodynamic target. The patient’s renal function at SUL-DUR initiation was normal (serum creatinine within the normal range for age) and remained stable throughout the treatment course.

**FIGURE 2:**
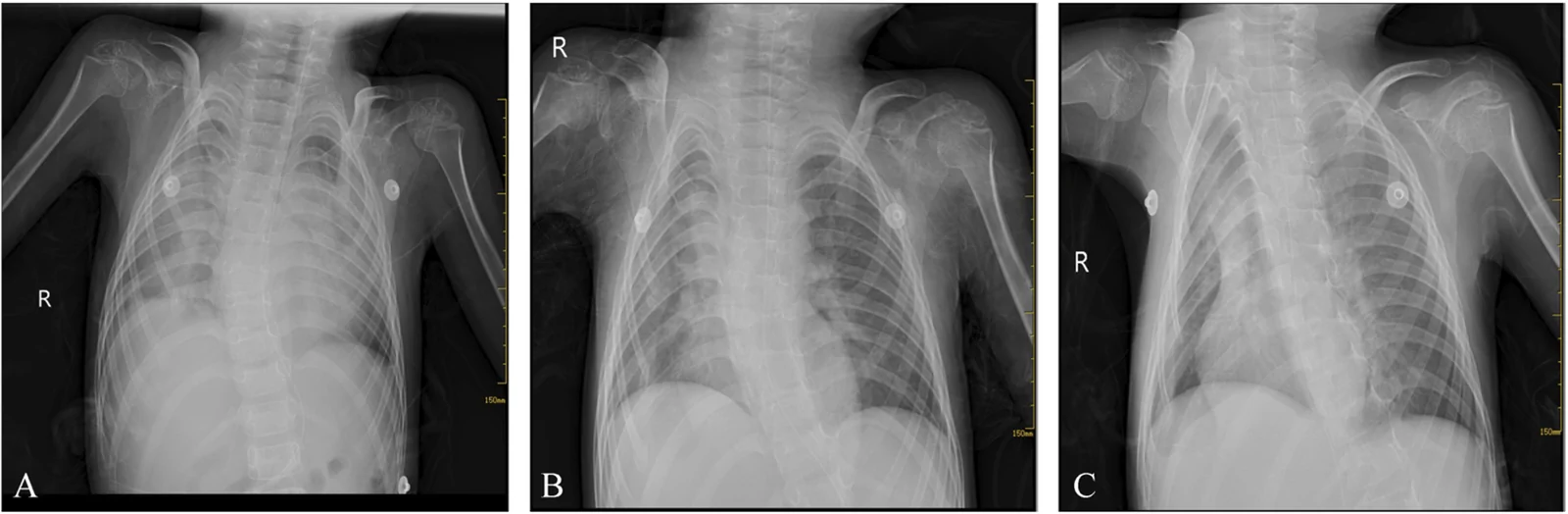
Chest radiographs showing progressive resolution of pulmonary infection. **(A)** Before SUL-DUR therapy, showing bilateral pulmonary infiltration. **(B)** Three days after initiation of SUL-DUR therapy, with noticeable improvement. **(C)** Ten days after initiation, showing substantial resolution of the infiltrates.

Within 24 h of SUL-DUR initiation, the patient’s peak temperature dropped from 38.8 °C to 38.0 °C with declining inflammatory markers; he became afebrile (36.9 °C) by 48 h. Table 1 summarizes the changes in laboratory parameters and clinical measurements before and after treatment. Chest imaging on day 23(3 days after SUL-DUR initiation) showed significant improvement compared with pre-treatment images (Figure 2). On day 32 of admission (after 12 days of SUL-DUR therapy), the patient was successfully transferred out of the PICU. No adverse events attributable to SUL-DUR were observed during the 12-day treatment or 6-week follow-up. Hepatic, renal, haematological, and neurological parameters remained stable, and no seizure exacerbation occurred despite the patient’s epilepsy.

**TABLE 1:** Laboratory parameters and temperature changes before and after SUL-DUR therapy.

Parameter	Pre-treatment	Day 2 post-treatment	Day 3 post-treatment	Day 7 post-treatment
WBC (×10^9^/L)	8.61	6.54	9.99	8.04
Neutrophils (%)	63.1	61.5	72.9	45.9
Lymphocytes (%)	24.5	22.1	15.8	41.7
Hemoglobin (g/L)	75	67*	96	90
Platelets (×10^9^/L)	472	413	523	599
CRP (mg/L)	50.37	30.64	5.42	4.26
PCT (ng/mL)	0.093	0.077	<0.02	<0.02
pH	7.434	7.417	7.420	7.441
PaO_2_ (mmHg)	86	90	97	124
PaCO_2_ (mmHg)	48.1	47.0	41	34.9
HCO_3_^−^ (mmol/L)	32.2	33.7	26.7	23.8
FiO_2_ (%)	50	50	40	35
PaO_2_/FiO_2_ ratio	172	180	242.5	354
Peak temperature (°C)	38.8	38.0	36.9	36.7

* The hemoglobin value on day 3 post-treatment was measured after transfusion of leukocyte-depleted suspended red blood cells.

## Discussion

CRAB is a critical-priority pathogen that causes refractory hospital-acquired pneumonia in critically ill and immunocompromised children (Sharma and Lakhanpal, 2025). Children with neurological comorbidities such as cerebral palsy and epilepsy are particularly prone to recurrent lower respiratory tract infections because of impaired airway clearance, prolonged immobilisation, repeated intubation or mechanical ventilation, and long-term antibiotic use (Zhang et al., 2023). These factors led to recurrent severe CRAB pneumonia in our patient, which responded poorly to multiple conventional combination regimens. To our knowledge, this is the first report of SUL-DUR use in a pediatric patient with cerebral palsy and epilepsy—a population that is simultaneously at heightened infection risk and disproportionately affected by economic barriers to novel antimicrobials.

The carbapenem resistance in this isolate was mediated by the blaOXA-23 gene, which encodes the OXA-23 class D β-lactamase. This enzyme is the most common carbapenemase globally distributed within the IC2 clonal lineage of *Acinetobacter* baumannii, and it efficiently hydrolyses carbapenems, penicillins and most cephalosporins, leading to broad-spectrum β-lactam resistance and severely limiting clinical treatment options (Guillén-Navarro et al., 2025). Our mNGS and antimicrobial susceptibility results were consistent with this mechanism: the isolate showed extensive resistance to all commonly used carbapenems, third- and fourth-generation cephalosporins, and fluoroquinolones. Only tigecycline, polymyxin B, minocycline and sulfonamides remained susceptible *in vitro*. Nevertheless, despite sequential and combination therapy with these seemingly susceptible agents (tigecycline, polymyxin B, minocycline and levofloxacin), the patient continued to experience fever, persistently elevated inflammatory markers, and progressive pulmonary infiltration on imaging. This clinical course strongly indicates that traditional single or combined anti-CRAB regimens have limited efficacy in children with severe underlying diseases and long-standing recurrent CRAB infection, highlighting the urgent need for novel therapeutic agents.

Sulbactam-durlobactam (SUL-DUR) is a novel dual β-lactamase inhibitor combination approved in China in 2024 specifically for drug-resistant *Acinetobacter* complex infections (Zai Lab and Innoviva Specialty Therapeutics, 2024). Unlike traditional β-lactam/β-lactamase inhibitor combinations, sulbactam is a penicillin derivative with intrinsic anti-Acinetobacter activity, but it is readily hydrolysed by β-lactamases encoded by contemporary isolates. Durlobactam, a diazabicyclooctane β-lactamase inhibitor, effectively inhibits class A, C and D serine β-lactamases (Karruli et al., 2023; McLeod et al., 2024). The synergistic action of the two components restores antibacterial activity against CRAB strains, directly targeting the core resistance mechanism present in this case. Although the official approved indication and standard dosing of SUL-DUR are limited to adult patients (≥18 years), its off-label use in this child was justified on compassionate grounds and the absence of effective therapeutic alternatives.

Given the family’s financial constraints, the patient initially received a reduced-dose SUL-DUR regimen supplemented with cefoperazone-sulbactam to achieve a total sulbactam dose of 150 mg/kg/day, which approaches the recommended sulbactam dose of 200 mg/kg/day for CRAB infections (Lockowitz et al., 2025). In a pediatric population pharmacokinetic model, a 4-h infusion of 50 mg/kg q6h (200 mg/kg/day) achieved ≥90% probability of target attainment (PTA) at an MIC of 8 μg/mL (target: 60% fT > MIC), while the lower dose of 25 mg/kg q6h (100 mg/kg/day) with 4-h infusion reached a breakpoint MIC of 4 μg/mL (Onita et al., 2025). Although our total sulbactam dose (150 mg/kg/day, 3-h infusion) falls between these two simulated regimens, direct extrapolation of PTA data should be made with caution given differences in total daily dose and infusion time. SUL-DUR was administered as a 3-h intravenous infusion every 6 h, consistent with the FDA-and National Medical Products Administration (NMPA)-approved package insert (*XACDURO®*) and the Infectious Diseases Society of America (IDSA) pediatric dosing guidance (Lockowitz et al., 2025). This prolonged infusion strategy is further supported by pediatric PK/PD simulation data (Onita et al., 2025).

After initiation of this individualized low-dose combination regimen (SUL-DUR plus cefoperazone-sulbactam), the patient rapidly defervesced, inflammatory markers declined significantly, pulmonary imaging improved markedly within a short time, and he was successfully transferred out of the PICU. This treatment outcome suggests that individualized dose adjustment of SUL-DUR together with rational combination therapy may achieve satisfactory results even in economically limited pediatric patients with severe refractory CRAB pneumonia. We do not advocate generalisation of this low-dose regimen to all pediatric patients. It represents a resource-adapted strategy for settings where the full dose is financially prohibitive. Individualised factors—including renal function, MIC, infection severity, and clinical response—must guide dosing decisions. Further pediatric PK studies of SUL-DUR are urgently needed. Future cases would also benefit from dedicated sulbactam MIC determination to enable formal PK/PD assessment.

A recently published pediatric case report described successful use of SUL-DUR in an infant with acute monocytic leukaemia and CRAB bacteremia with cutaneous involvement (Fu et al., 2025). The present case extends the pediatric evidence base in four important respects. First, our patient had chronic neurological comorbidities (cerebral palsy and epilepsy) rather than haematological malignancy, expanding the population for which SUL-DUR may be considered. Second, the infection site was recurrent VAP rather than bacteremia with cutaneous involvement, demonstrating efficacy across different CRAB infection phenotypes. Third, and most critically, we employed a reduced-dose regimen driven by economic necessity—a real-world scenario not previously documented—suggesting that SUL-DUR may retain clinical efficacy even at doses below the standard adult regimen when combined with a second sulbactam-containing agent. Fourth, this case contributes valuable safety information: no SUL-DUR-related adverse events were observed during the 12-day treatment or the 6-week follow-up, and importantly, no seizure exacerbation occurred despite the patient’s underlying epilepsy. Together, these two cases provide complementary evidence supporting the use of SUL-DUR across different pediatric populations with CRAB infections.

Several limitations warrant acknowledgment. First, SUL-DUR plasma concentrations were not measured, precluding formal PK/PD confirmation of target attainment. Second, as a single case, the generalizability of our findings is inherently limited. Third, the contribution of cefoperazone-sulbactam to the observed clinical response cannot be isolated from that of SUL-DUR. Fourth, microbiological clearance was not documented by repeat culture or mNGS, as the patient’s rapid clinical improvement rendered repeat bronchoscopy clinically unwarranted and follow-up mNGS was unavailable in our resource-limited setting. The absence of microbiological response data limits the assessment of definitive pathogen eradication and should be addressed in future prospective studies. Fifth, sulbactam-alone MIC was not determined for this isolate, precluding formal PK/PD target attainment analysis. Although the Onita et al. (2025) pediatric PK data provide a quantitative framework for estimating target attainment, direct extrapolation to our lower-dose, shorter-infusion regimen is limited. Despite these constraints, the temporal association between SUL-DUR initiation and rapid clinical improvement—after failure of multiple prior regimens—provides supportive evidence for its efficacy. Formal pediatric pharmacokinetic studies and prospective trials are urgently needed.

### Patient perspective

The patient’s legal guardian provided written informed consent for publication of this case report and accompanying images. The family expressed deep gratitude for the successful treatment outcome and agreed to share their experience in the hope of helping other children with similar life-threatening infections.

## Conclusion

This case demonstrates that SUL-DUR, even at a reduced dose when combined with a second sulbactam-containing agent, was associated with a rapid clinical response in critically ill children with refractory CRAB pneumonia. In resource-limited settings where the full adult dose is financially prohibitive, an individualized low-dose strategy guided by PK/PD principles and supplemented by combination therapy warrants further investigation as a pragmatic treatment approach. Larger prospective studies are needed to establish optimal pediatric dosing and confirm generalisability.

## Data Availability

The original contributions presented in the study are included in the article/supplementary material, further inquiries can be directed to the corresponding authors.
